# Culture System and Nutrient Restriction Shape Antioxidant Activity in *In Vitro* Spearmint (*Mentha spicata* L.) Shoots

**DOI:** 10.3390/plants14243863

**Published:** 2025-12-18

**Authors:** Raquel Martínez-Carrillo, Fátima Z. Behloul, María Á. Ferrer, Antonio A. Calderón

**Affiliations:** Department of Agronomic Engineering, Universidad Politécnica de Cartagena, Paseo Alfonso XIII 48, 30203 Cartagena, Spain; raquel.martinez@edu.upct.es (R.M.-C.); fatima.bkourdache@edu.upct.es (F.Z.B.); mangeles.ferrer@upct.es (M.Á.F.)

**Keywords:** spearmint, antioxidant activity, rosmarinic acid, *in vitro*-shoot cultures, antidiabetic potential, phenolic compounds

## Abstract

Spearmint phytochemicals exhibit remarkable antidiabetic, antioxidant, and broad pharmacological activities. *In vitro* organ cultures offer an efficient and sustainable platform for enhancing the production of these bioactive metabolites, although optimized media and cultivation strategies are essential to maximize yields. Here, four Murashige and Skoog (MS) medium variants and three cultivation systems—agar-solidified, static-liquid (L), and agitated-liquid (LA)—were evaluated to assess phenolics, antioxidant capacity, antidiabetic potential, and metabolic biochemical markers in *in vitro*-grown spearmint shoots. Half-strength MS (MS/2) consistently produced the highest antioxidant activity and accumulation of phenolics and sugars across all systems. The MS/2–L combination markedly boosted antioxidant responses, increasing 2,2-diphenyl-1-picrylhydrazyl (DPPH) values up to 27-fold and ferric reducing antioxidant power (FRAP) values tenfold relative to full-strength MS. Antioxidant capacity strongly correlated with total phenolics, flavonoids, rosmarinic acid, antidiabetic activity, and carbohydrate levels. Lipid peroxidation analysis further revealed that shoots cultured under LA conditions showed the highest level of malondialdehyde (MDA) accumulation, whereas MSN/2 (half-strength nitrogen) consistently yielded the lowest MDA levels across all cultivation systems. Collectively, these results highlight the strong influence of nutrient availability and culture system on the metabolic performance of *in vitro*-grown spearmint. In conclusion, nutrient limitation combined with liquid cultivation effectively enhances antioxidant metabolite accumulation, providing valuable criteria for the future design and optimization of scalable bioreactor systems.

## 1. Introduction

Aromatic and medicinal plants are currently the focus of intensive research as sustainable and cost-effective sources of bioactive compounds with antimicrobial, antioxidant, anti-inflammatory, and antitumor properties [1,2]. This growing interest is driven by the demand for eco-friendly alternatives to synthetic compounds, the need to address antimicrobial resistance, and strong evidence linking plant-derived compounds to improved human and animal health and well-being [3].

Spearmint (*Mentha spicata* L.) is an aromatic perennial herb from the Lamiaceae family, widely recognized for its significant economic value and extensive use in food, cosmetics and medicine [4,5,6,7,8]. Spearmint has been reported to exhibit chemopreventive and antimutagenic properties [9], act as an analgesic in patients with osteoarthritis [10], and alleviate gastrointestinal disorders such as irritable bowel syndrome [11]. It has also shown high insecticidal potential against vector pests [12,13], antifungal [14], and antibacterial [8,15] activities.

The diverse biological activities of spearmint are closely linked to its chemical composition. Spearmint essential oil is particularly rich in oxygenated monoterpenes, including menthol, carvone, and pulegone, while the non-volatile fractions contain abundant hydroxycinnamic acid derivatives and flavonoids [6]. These compounds play a crucial role in mediating the plant’s wide range of biological effects [6]. The accumulation of bioactive compounds in plants strongly depends on genetic background, environmental factors and agronomic practices. Many of these high-value compounds are synthesized as adaptive responses to biotic or abiotic stress [16,17].

*In vitro* culture provides a reliable and versatile platform for producing plant bioactive compounds, enabling the simulation of stress conditions that enhance their biosynthesis [18]. Moreover, variation in the media composition can stimulate the synthesis and accumulation of specialized metabolites significantly affecting physiological processes essential for plant growth and biomass formation [19]. There is no doubt that the type, concentration, and balance of mineral nutrients in culture media significantly affect the accumulation of specialized metabolites. However, the way this occurs is often species- and metabolite-specific and depends on interactions with other environmental factors. For instance, high levels of macronutrients (N, P, and K) in *Prunella vulgaris* culture media led to increased accumulation of total soluble phenols and rosmarinic acid [20]. In contrast, nutrient limitation in *Ocimum basilicum* culture media of different cultivars resulted in increased synthesis of phenolic compounds [21].

The consistency of the culture medium is another factor that can significantly influence the growth and production of commercially interesting compounds from *in vitro* cultures of plant materials. For example, *Spiraea betulifolia* cultures in a liquid medium showed greater growth and accumulation of phenolic compounds than cultures in a gelatinized medium [22]. This is crucial because, in order to exploit *in vitro* cultures commercially for biomass and/or specialized metabolite production, scaling up is necessary, which involves using liquid culture media and bioreactors. Advantages of using a liquid medium for micropropagation include easier handling of cultures (inoculation and removal), saving labor and time, and constant contact of the cultures with the medium, facilitating nutrient absorption and stimulating growth. However, liquid cultures have disadvantages as well. Asphyxia, hyperhydricity, and physiological disorders can occur in submerged cultures [23].

Although several studies have demonstrated the effects of fertilization regimes on bioactive phytochemicals in various medicinal and aromatic plant species [24,25,26,27,28,29,30], fewer have investigated the combined influence of basal medium composition and consistency on *in vitro*-grown plants. This study examines how spearmint shoots respond to four culture media with different macro- and micronutrient compositions. The media are applied to three cultivation systems: agar-solidified, static liquid, and agitated liquid. The media are derived from the basal medium described by Murashige and Skoog in 1962 and include full-strength MS (MS), half-strength MS (MS/2), MS with half the amount of macronutrients and iron (M/2S), and MS with half the concentration of potassium nitrate and ammonium nitrate (MSN/2).

The objective of this study was to identify the most suitable combination of nutrient formulation and cultivation system to optimize the accumulation of bioactive compounds in spearmint shoots without significantly affecting growth performance. The results obtained in the study could provide insight into the feasibility of using this plant material as a source of high value-added metabolites in large-scale cultures. They may also suggest factors to consider when selecting an appropriate bioreactor.

## 2. Results

### 2.1. Effect of Basal Media Composition and Culture System on the Content of Chlorophylls, Soluble Sugars and Starch

**Figure 1:**
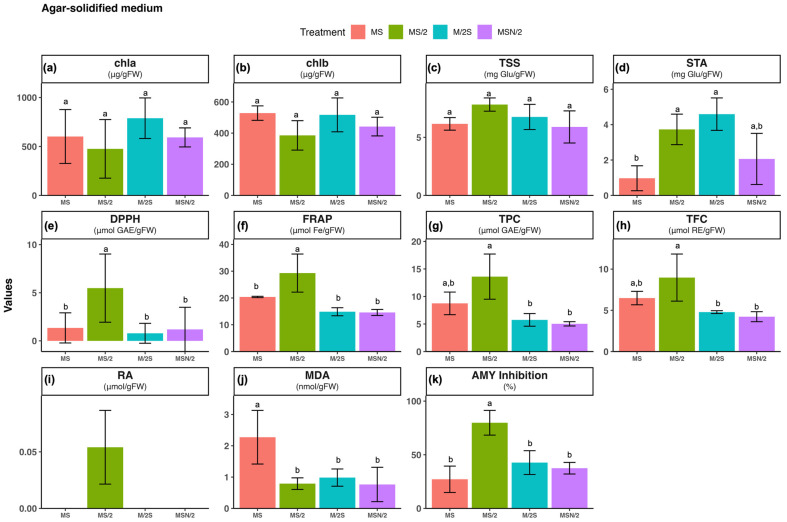
Effect of basal medium composition on (**a**) chlorophyll a, (**b**) chlorophyll b, (**c**) total soluble sugars, (**d**) starch, (**e**) DPPH radical scavenging activity, (**f**) ferric reducing antioxidant power, (**g**) total phenolic content, (**h**) total flavonoid content, (**i**) rosmarinic acid, (**j**) malondialdehyde, and (**k**) α-amylase inhibitory activity in *in vitro*-grown spearmint shoots cultivated in agar-solidified media. Data represent means ± SD of two independent experiments. Different letters indicate significant differences according to Tukey’s HSD test at *p* ≤ 0.05. Abbreviations: MS, full-strength Murashige and Skoog medium; MS/2, half-strength MS medium; M/2S, MS with diluted (1/2) macronutrients and iron elements; MSN/2, MS with diluted (1/2) nitrate and ammonium concentration.

**Figure 2:**
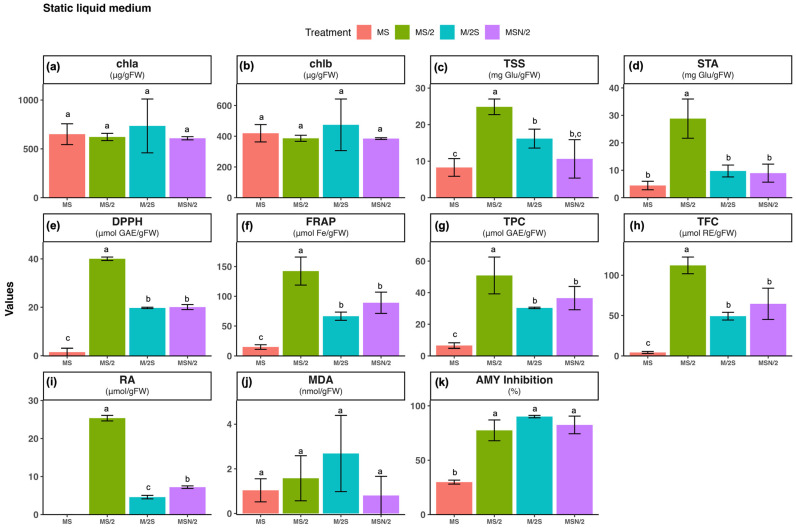
Effect of basal medium composition on (**a**) chlorophyll a, (**b**) chlorophyll b, (**c**) total soluble sugars, (**d**) starch, (**e**) DPPH radical scavenging activity, (**f**) ferric reducing antioxidant power, (**g**) total phenolic content, (**h**) total flavonoid content, (**i**) rosmarinic acid, (**j**) malondialdehyde, and (**k**) α-amylase inhibitory activity in *in vitro*-grown spearmint shoots cultivated in liquid static media. Data represent means ± SD of two independent experiments. Different letters indicate significant differences according to Tukey’s HSD test at *p* ≤ 0.05. Abbreviations: MS, full-strength Murashige and Skoog medium; MS/2, half-strength MS medium; M/2S, MS with diluted (1/2) macronutrients and iron elements; MSN/2, MS with diluted (1/2) nitrate and ammonium concentration.

**Figure 3:**
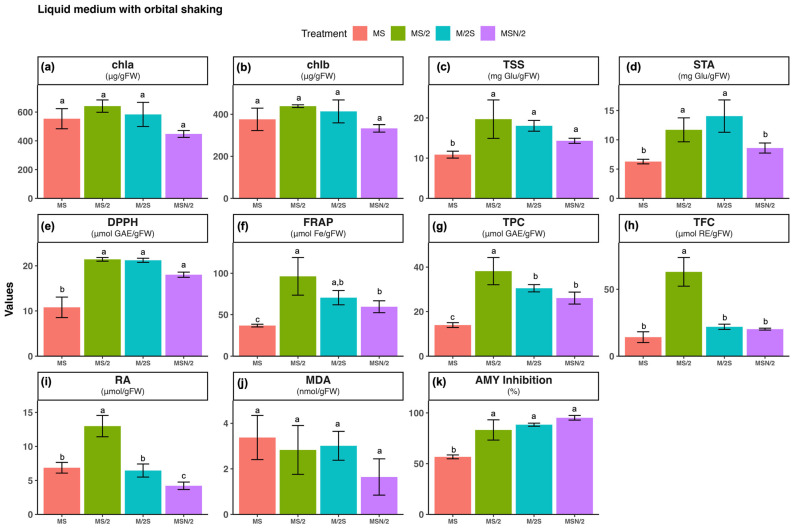
Effect of basal medium composition on (**a**) chlorophyll a, (**b**) chlorophyll b, (**c**) total soluble sugars, (**d**) starch, (**e**) DPPH radical scavenging activity, (**f**) ferric reducing antioxidant power, (**g**) total phenolic content, (**h**) total flavonoid content, (**i**) rosmarinic acid, (**j**) malondialdehyde, and (**k**) α-amylase inhibitory activity in *in vitro*-grown spearmint shoots cultivated in agitated liquid media. Data represent means ± SD of two independent experiments. Different letters indicate significant differences according to Tukey’s HSD test at *p* ≤ 0.05. Abbreviations: MS, full-strength Murashige and Skoog medium; MS/2, half-strength MS medium; M/2S, MS with diluted (1/2) macronutrients and iron elements; MSN/2, MS with diluted (1/2) nitrate and ammonium concentration.

No significant differences were observed in the contents of chlorophyll a and b among the different basal media (see Supplementary Table S1) or culture systems tested (panels a and b in Figure 1, Figure 2 and Figure 3). In contrast, marked variations were detected in total soluble sugar (TSS) and starch (STA) contents (panels c and d in Figure 1, Figure 2 and Figure 3). TSS accumulation was more than twofold higher in all modified Murashige and Skoog (MS) formulations under liquid culture conditions (both static and agitated) compared with their corresponding agar-solidified media, with the greatest increase observed in the static liquid MS/2 medium (~3.2-fold). Similarly, STA content was also higher (>3-fold) in all the modified MS liquid media than in the solid ones, with the most pronounced change in the static liquid MS/2 medium (~7.4-fold). The two-way ANOVA results indicated that TSS and STA contents were significantly influenced by the basal medium composition and the culture system, as well as by their interaction (*p* ≤ 0.001) (Supplementary Table S2).

### 2.2. Effect of Basal Media Composition and Culture System on Total Antioxidant Activity and Accumulation of Phenolic Compounds

The total antioxidant activity of spearmint ethanolic extracts was evaluated using the DPPH (panel e) and FRAP (panel f) assays. The highest antioxidant activity was observed in spearmint shoots cultured in MS/2 medium across all culture systems tested. Overall, for the three modified MS media, antioxidant activity was consistently higher under static liquid culture conditions compared with the other systems, as shown in panels e and f of Figure 1, Figure 2 and Figure 3. Notably, the combination of MS/2 medium and static liquid culture produced the strongest antioxidant response, with values exceeding those obtained in full-strength MS medium by up to 27-fold (DPPH) and tenfold (FRAP).

Similarly, the highest total phenol content (TPC, panel g) and total flavonoid content (TFC, panel h) were also recorded in the MS/2 medium across all culture systems tested, with the greatest increases observed in the static liquid MS/2 medium (~4-fold and ~13-fold, respectively), followed by the agitated culture (~3-fold and ~7-fold, respectively) when compared with the agar-solidified medium. The two-way ANOVA results revealed that antioxidant activity (DPPH and FRAP), and phenolic contents (TPC and TFC) were significantly affected by both the basal medium composition and the culture system, as well as by their interaction (*p* ≤ 0.001) (Supplementary Table S2).

### 2.3. Effect of Basal Media Composition and Culture System on Phenolic Profile and Rosmarinic Acid (RA) Content

**Figure 4:**
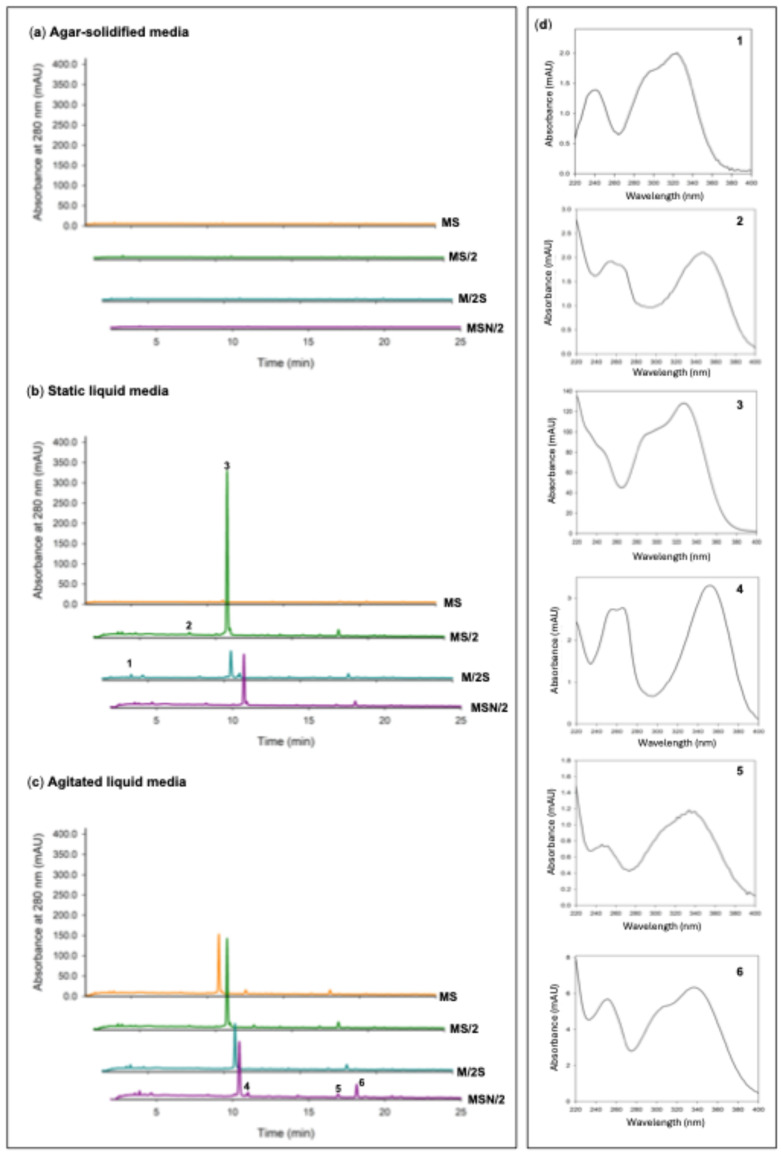
UPLC chromatograms recorded at 280 nm of ethanolic extracts of *in vitro*-grown spearmint shoots in (**a**) agar-solidified media, (**b**) liquid static media, and (**c**) agitated liquid media The UV spectra of the six significant peaks in the chromatograms (**d**). Peak 1, caffeic acid; peak 3, rosmarinic acid; peaks 2 and 4, not identified flavonoid derivatives; peaks 5 and 6, not identified hydroxycinnamic acid derivatives. Abbreviations: MS, full-strength Murashige and Skoog medium; MS/2, half-strength MS medium; M/2S, MS with diluted (1/2) macronutrients and iron elements; MSN/2, MS with diluted (1/2) nitrate and ammonium concentration.

Given the marked increases observed in both total phenolic and flavonoid contents, the phenolic profile of the spearmint ethanolic extracts was further investigated by UHPLC analysis. The chromatograms at 280 nm, particularly those obtained from shoots grown under liquid culture conditions, were characterised by the presence of a prominent peak at a retention time of approximately 10.4 min (peak 3), which was identified as rosmarinic acid (RA) based on the coincidence of its retention time and UV spectrum with those of the reference standard. In addition to this major peak, five smaller peaks were detected (peaks 1 and 2 with tR of of approximately 4 and 8.5 min, respectively and peaks 4, 5 and 6 at retention times of approximately 11, 17, and 18 min, respectively). Interestingly, chromatograms from shoots grown on agar-solidified media showed almost no visible peaks (Figure 4).

Considering that the main peak of the chromatograms corresponded to RA, its quantification, determined by integration of the peak area in the chromatogram at 325 nm, is presented in panel i of Figure 1, Figure 2 and Figure 3. As can be observed, with the exception of MS/2 medium, RA was under the limit of detection in any of the other basal media formulations under agar-solidified conditions. Under liquid culture conditions, the highest RA content was observed in the MS/2 medium, with the greatest levels in the MS/2 static liquid medium in both systems (23.4 ± 0.7 μmol/g FW and 13.0 ± 1.6 μmol/g FW, for static and agitated liquid medium, respectively). Again, the two-way ANOVA results indicated that RA contents were significantly influenced by the basal medium composition and the culture system, as well as by their interaction (*p* ≤ 0.001) (Supplementary Table S2).

### 2.4. Effect of Basal Media Composition and Culture System on Lipid Peroxidation

The extent of lipid peroxidation was evaluated as a marker of oxidative damage (panel j in Figure 1, Figure 2 and Figure 3). The results revealed that the malondialdehyde (MDA) content was higher in spearmint shoots cultured under agitated liquid conditions with the highest value observed in the MS medium. In contrast, the lowest MDA levels were recorded in shoots grown in MSN/2 across all the cultures systems. The two-way ANOVA results indicated that MDA content was significantly affected by the basal medium composition (*p* ≤ 0.001) and the culture system (*p* ≤ 0.01), whereas no significant interaction was detected between these two factors (Supplementary Table S2).

### 2.5. Effect of Basal Media Composition and Culture System on the α-Amylase Inhibitory Activity

The analysis of the α-amylase inhibitory activity (panel k in Figure 1, Figure 2 and Figure 3) showed that the highest inhibitory activity was obtained in extracts from spearmint shoots grown under liquid culture conditions (both static and agitated), with the greatest inhibition observed in the agitated liquid MSN/2 medium in both systems (95.2 ± 2.3% and 90.1 ± 1.1%, respectively). Again, the two-way ANOVA results indicated that α-amylase inhibitory activities were significantly influenced by the basal medium composition and the culture system, as well as by their interaction (*p* ≤ 0.001) (Supplementary Table S2).

### 2.6. Principal Component Analysis

**Figure 5:**
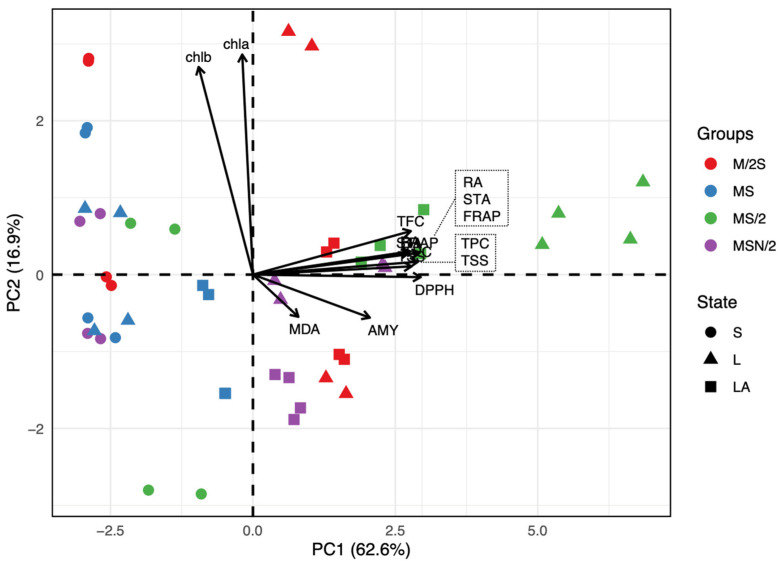
Principal component analysis (PCA) biplot based on the correlation matrix of biochemical parameters measured in *in vitro*-grown spearmint shoots cultivated in four MS medium variants across three cultivation systems. Abbreviations: AMY, α-amylase inhibitory activity; chl, chlo-rophyll; DPH, DPPH radical scavenging activity; FRAP, ferric reducing antioxidant power; MDA, malondialdehyde; RA, rosmarinic acid; STA, starch content; TFC, total flavonoid content; TPC, total phenol content; TSS, total soluble sugars; S, agar-solidified; L, liquid static; LA, agitated-liquid.

A principal component analysis (PCA) was conducted to visualize data trends and to detect possible clusters within samples. The PCA revealed that the first two components explained 79.5% of the total variance (PC1 = 62.6%, PC2 = 16.9%), indicating a strong dimensionality reduction. The biplot (Figure 5) shows a clear separation of samples along PC1, which is primarily associated with antioxidant capacity (DPPH and FRAP), phenolics (TPC, TFC and RA) and soluble sugars (TSS) and starch (STA), all projecting strongly in the positive direction of PC1. Conversely, chlorophyll-related variables (chla and chlb) exhibit high positive loadings on PC2, suggesting that this component captures variation related to pigment content rather than antioxidant capacity.

The grouping of samples by culture media (M/2S, MS, MS/2, MSN/2) and medium consistency (S, L, LA) indicates distinct clustering patterns. Samples from MS/2 (green triangles) are positioned toward the positive side of PC1, reflecting higher antioxidant activity and phenolic content. In contrast, samples from M/2S (red circles) and MSN/2 (purple squares) cluster closer to the origin or negative PC1 values, suggesting lower antioxidant-related attributes. PC2 further differentiates samples based on chlorophyll content, with some MS and M/2S samples exhibiting higher pigment levels.

## 3. Discussion

Profitably producing biomass and/or metabolites of commercial interest from *in vitro* cultures of plant materials necessarily involves scaling up the cultures and automating the processes as much as possible. Bioreactors are used to achieve this. In the case of plants, they can house either disorganized cultures (cell cultures) or organized cultures (organ or whole plant cultures). In either case, working with liquid culture media is practically essential. Liquid culture media offer several advantages that lower production costs. These advantages include eliminating the use of gelling agents (one of the most expensive culture medium constituents), easier handling of cultures, and a more homogeneous and rapid response from the cultivated materials. However, not all plant materials tolerate cultivation in liquid media adequately. Hypoxia or anoxia, which can occur in this type of culture, can trigger various physiological disorders, such as hyperhydricity syndrome [23]. These disorders negatively affect the growth and metabolism of the cultures. For these reasons, it is necessary to test the performance of plant material in liquid culture media on a laboratory scale before moving on to commercial-scale cultivation. This study analyzes how spearmint shoot cultures respond to transitioning from gelatinized to liquid culture media, with or without agitation. Additionally, it examines how changes in medium composition and the interaction between medium composition and consistency affect the accumulation of bioactive compounds in spearmint’s aerial tissues.

Chlorophyll content is a widely used indicator for assessing the physiological status of *in vitro* shoot cultures. In the present study, no significant differences in chlorophyll a and b levels were detected among the tested treatments (see Supplementary Table S2), suggesting that variations in medium composition and culture conditions did not substantially alter the vigor of the cultures. However, these results contrast with those of [20], who found that reducing the nutrient concentration in the MS medium by half or a quarter significantly decreased total chlorophyll levels and the chlorophyll a/b ratio in *Prunella vulgaris* shoots grown *in vitro*. According to these authors, a nitrogen deficit in culture media has been demonstrated to inhibit chlorophyll synthesis and promote its degradation. The nitrogen levels in MS medium are notably elevated (see Supplemental Table S1). These levels are estimated to be approximately 80 times higher than the levels typically found in agricultural soils [31]. In the present study, the reduction of nitrogen levels in the culture media to half their original concentration was found to be ineffective in inducing a nutritional deficit that would result in a substantial alteration of the physiological state of spearmint shoots during the cultivation period.

Although chlorophyll content is a valuable indicator of physiological status in *in vitro* shoot cultures, it should be interpreted alongside other growth and quality parameters for a comprehensive assessment. The reliability of chlorophyll content varies with species, genotype, and culture conditions. For this reason, other biochemical markers were analyzed that could provide insight into the physiological state of the *in vitro*-cultured material. The analysis of TSS and STA showed a different pattern depending on the composition and consistency of the culture medium, with significant interaction between both factors (Supplementary Table S2). Shoots incubated in liquid media had higher TSS and STA values, which may be related to greater accessibility to nutrients, including sucrose, in the medium (panels c and d in Figure 1, Figure 2 and Figure 3). In terms of the effect of nutrient levels, shoots grown in MS are among those with the lowest TSS and STA contents in any culture system tested, while those incubated in MS/2 are always among those with the highest sugar levels. Interestingly, a strong correlation was found between TSS and STA levels and antioxidant capacity and phenolic compound levels in spearmint tissues (Figure 5 and Supplementary Figure S1). This suggests a connection between these parameters.

Sugars are involved in physiological functions that extend beyond the production of metabolic energy and structural roles. In fact, we are learning more and more about the regulatory function of these compounds on genes related to growth, development, metabolism, and stress resistance [32]. Sugars appear to play a significant role in the cellular response to oxidative stress via various mechanisms. They can directly deactivate reactive oxygen species (ROS) and induce metabolic pathways that lead to the synthesis of primary and secondary antioxidants [32]. For instance, sucrose accumulation has been shown to help maintain redox balance in postharvest fruits by neutralizing ROS and stimulating ascorbic acid synthesis [33]. In the present study, the observation that the highest levels of TSS and STA are detected in shoots where, a priori, conditions would be expected to be more stressful, i.e., in the MS/2 medium and in the L culture system (characterized by lower nutrient levels and reduced aeration), could be indicative of a heightened activation of the defensive response to stress in comparison to the other culture conditions.

The pentose phosphate pathway is one of the metabolic pathways stimulated by sugars. This pathway is essential for maintaining redox homeostasis because it produces NADPH, which is necessary for enzymes that maintain the pool of cellular reductants [34]. The pentose phosphate pathway has been demonstrated to supply the precursors necessary for the synthesis of phenolic compounds, which have been shown to act as antioxidants. This action can occur in two distinct ways: firstly, by directly neutralizing ROS, and secondly, by acting as electron donors in reactions catalyzed by the peroxidase enzyme, which leads to the elimination of H_2_O_2_ [35]. In situations involving stress, an accumulation of sugars and phenols has been observed within the vacuoles. Consequently, the hypothesis has been put forth that these compounds may constitute a component of a redox buffering system, the function of which would be to neutralize the excess of ROS produced within the cytoplasm [32]. This study found the highest accumulation of phenolic compounds, antioxidant capacity, and sugar levels in spearmint shoots grown in MS/2 media and the L culture system. This could reflect enhanced activation of the vacuolar antioxidant system. Similar results of the co-accumulation of sugars and phenols have recently been reported in other *in vitro* culture systems [36].

Rosmarinic acid (RA), one of the most abundant phenolic compounds in the aerial parts of Lamiaceae family plants, has great therapeutic potential due to its anticancer, anti-inflammatory, antidiabetic, antimicrobial, and antiviral properties. These properties are partly associated with its strong antioxidant properties [37]. This compound was found to be the most abundant in the spearmint shoot extracts analyzed in this study (Figure 4), exhibiting the highest levels in shoots incubated in MS/2 culture media and in the L culture system (panel i, in Figure 1, Figure 2 and Figure 3). Previous studies on nutrient limitation in *in vitro* cultures of spearmint using MS and MS culture media with reduced nutrient levels (by half or a quarter) have revealed an inverse relationship between nutrient levels and the accumulation of phenolic compounds. This behavior is explained by competition between primary and secondary metabolism when nutrients are adequately supplied [38]. Other studies carried out with different Lamiaceae species cultivated *in vitro* have shown that nutrient limitation generally increases the levels of secondary metabolites and specifically increases the levels of phenolic compounds, including (RA) [39,40,41]. These studies associated the increase in phenol levels with deficiencies in Mg or Fe content in *Thymus lotocephalus* and *Lavandula viridis* cultures [39]; N, Mg, or K content in *Perilla frutescens* cultures [41]; or a 50% reduction in macro- and micronutrient concentration in *Origanum vulgare* cultures [40]. They also explained the increase in phenol levels by oxidative stress induced in the crops by nutrient limitation. Although the present study did not assess the retention of enhanced antioxidant and phenolic profiles after *ex vitro* acclimatization, previous research offers insight into the potential transient physiological adjustments undergone by micropropagated plants upon transfer to greenhouse conditions. These adjustments may involve modifications to the antioxidant systems of the plants [42]. Research conducted on micropropagated *Mentha* species, including *M. spicata*, has demonstrated that *in vitro*-raised plantlets transferred to *ex vitro* conditions exhibit elevated levels of total phenolic content. This phenomenon may be indicative of at least a transient maintenance of their biosynthetic capabilities [43].

Levels of MDA, which are derived from lipid peroxidation, can be considered a marker of oxidative stress in tissues. However, a positive correlation is not always found between this parameter and the accumulation of phenolic compounds, which are considered another marker of nutritional stress [44]. In *T. lotocephalus*, for example, MDA, TPC, TFC, RA, and antioxidant capacity assays showed a weak, non-significant positive correlation. In *L. viridis*, however, these parameters were inversely related [39]. A negative correlation between phenolic compounds and MDA is considered proof that these compounds effectively protect *O. basilicum* from stress caused by nutrient limitation [21]. In our study, MDA levels were affected by the composition and consistency of the culture medium. However, no interaction between these two factors was observed (see Supplementary Table S2) and only a weak positive correlation was found between MDA levels and TPC, TFC, RA, FRAP, and DPPH levels (see Supplementary Figure S1). These results suggest that the production of antioxidant compounds by spearmint tissues may be sufficient to prevent widespread oxidative damage.

In addition to RA, other phenolic compounds were detected in the extracts of spearmint. Both caffeic acid and the derivatives of hydroxycinnamic acids and unidentified flavonoids (Figure 4) are expected to have antioxidant activity and, in general, significant biological activity. As found in other studies [38,39], the correlation between antioxidant capacity tests and TPC, TFC, and RA levels is very high, suggesting that these compounds are primarily responsible for this capacity in the tissues of *M. spicata* and other species.

The properties of RA and other phenolic compounds include a wide spectrum of biological activities. One of the most interesting therapeutic applications of RA is its use in the treatment of diabetes, through an effect mediated by reducing the expression of the enzyme phosphoenolpyruvate carboxykinase and increasing the expression of the glucose transporter GLUT4 [45]. Another mechanism by which RA and other phenolic compounds can control blood glucose levels is through the inhibition of the pancreatic enzyme α-amylase (AMY) [46]. In our study, it was observed that extracts from shoots grown in liquid media, and in particular those with nutrient limitation, showed greater AMY inhibitory activity, which is in line with the RA, TPC, and TFC levels found.

In summary, the utilization of liquid media and the implementation of controlled nutritional deficits can be regarded as a promising strategy for enhancing the levels of bioactive compounds in tissues cultured *in vitro*. However, because all experiments were conducted using a single genotype, the possibility of genotype × environment interactions cannot be ruled out. Therefore, caution should be exercised in generalizing these results, and future studies should include additional genotypes to assess the broader applicability of this approach and to further validate its potential for metabolite production at larger scales.

## 4. Materials and Methods

### 4.1. Plant Material and Culture Conditions

Explants of *M. spicata* subsp. *spicata* L. were obtained from a mother plant provided by a commercial greenhouse (Vergel Garden y Servicios S.L., Cartagena, Spain). Spearmint clonal shoots were grown on a modified Murashige & Skoog (MS) medium with half-strength NH_4_NO_3_ and KNO_3_ (MSN/2) (Catalog #M 0236, Dufecha-Biochemie, Haarlem, The Netherlands) supplemented with casein hydrolysate (250 mg L^−1^), sucrose (3% *w*/*v*) and 0.8% Difco Bacto agar and maintained by monthly subcultures. Cultures were kept at 25 °C under a 16 h light/8 h dark photoperiod and a photon flux density of 100 μmol m^−2^ s^−1^ (Sanyo, versatile environmental test chamber, MLR-351H, Osaka, Japan).

For this study, four-week-old *in vitro*-grown spearmint shoots were cut into nodal segments containing a single node. Three explants were placed in 100-mL Erlenmeyer flasks containing 20 mL of culture medium. Cultivation was performed on four modified MS media: full-strength (MS), half-strength (MS/2), MS with half-strength macronutrients and iron (M/2S), and MS with half-strength NH_4_NO_3_ and KNO_3_ (MSN/2). All media were supplemented with casein hydrolysate (250 mg L^−1^) and sucrose (3% *w*/*v*). Agar-solidified media additionally contained 0.8% Difco Bacto agar. Cultures were maintained at 25 °C under a 16 h light/8 h dark photoperiod and a photon flux density of 100 μmol m^−2^ s^−1^. Agitated liquid cultures were placed on an orbital shaker set at 80 rpm. After four weeks of growth, shoots from the three culture systems assayed (agar-solidified, static liquid, and agitated liquid cultures; Supplementary Figure S2) were snap-frozen in liquid nitrogen, ground to a fine powder, and stored at −80 °C until further analysis. The experiment was conducted twice, each time including two biological replicates per treatment, with each replicate consisting of five pooled *in vitro*-cultured shoots. All cultures were grown under the same temperature and light conditions detailed previously.

### 4.2. Metabolic Markers Extraction

For the analysis of metabolic markers, liquid nitrogen-powdered shoots (~100 mg) were extracted twice with ice-cold 80% ethanol (0.7 mL) using sonication followed by centrifugation at 15,000× *g* for 15 min at 4 °C, as previously described [47]. The combined supernatants were used for the spectrophotometric quantification of chlorophyll a and b, total soluble sugars (TSS), total phenolic content (TPC), total flavonoid content (TFC) and malondialdehyde (MDA) levels, and the resulting pellets for starch (STA) determination according to [48].

### 4.3. Total Antioxidant Capacity

The total antioxidant capacity of the spearmint ethanolic extracts was evaluated using the DPPH (2,2-diphenyl-1-picrylhydrazyl radical) and FRAP (ferric reducing antioxidant power) assays [49,50,51]. DPPH radical scavenging activity was quantified at 517 nm and expressed as micromoles of gallic acid equivalent (GAE) reduced per gram of fresh weight (FW), calculated from a calibration curve in the range 10–500 μM. FRAP was expressed as micromoles of Fe(II) per gram FW, calculated from a FeSO_4_·7H_2_O calibration curve in the range 25–1500 μM.

### 4.4. α-Amylase Inhibition Assay

The α-amylase inhibitory assay was performed using 2-chloro-4-nitrophenyl α-D-maltotrioside (CNPG3) as a substrate, as described by [52], with minor modifications. Prior to the assay, ethanolic spearmint extracts (200 µL) were evaporated to dryness under a nitrogen stream and resuspended in 80 µL of Milli-Q water by vortexing. Briefly, 20 µL of each aqueous extract was loaded in 96-well microtiter plate, followed by the addition of 80 µL of α-amylase solution (1.5 U/mL, prepared in 50 mM phosphate buffer, pH 6.9 containing 6 mM NaCl). The plate was incubated at 30 °C for 15 min, after which 100 µL of CNPG3 (355 µM dissolved in the same phosphate buffer) was added to each well. The reaction mixtures were incubated at 30 °C for 5 min, and absorbance was recorded at 405 nm before and after incubation using a microplate reader (Multiskan GO, Thermo Scientific, Vantaa, Finland). Milli-Q water (20 µL) was used as a blank control, and acarbose (5–10 µg/mL) served as the positive control. All the experiments were performed in triplicate. The α-amylase inhibitory activity was expressed as percentage inhibition (%) based upon initial velocity and was calculated as: Inhibition (%) = 100 − [(OD/min of extract)/(OD/min of control)] × 100.

### 4.5. UHPLC-DAD Analysis

For identification and quantification of individual phenols, an ultra-performance liquid chromatography system (Agilent 1290 Infinity II LC system; Agilent Technologies, Santa Clara, CA, USA) equipped with a G7167B multisampler, a G7104A quaternary pump, a G7116B column heater, and a G7117B diode array detector (DAD) was used. Separations were performed in a ZORBAX Eclipse Plus C18 RRHD analytical column (50 × 2.1 mm, 1.8 μm, Agilent Technologies, Santa Clara, CA, USA). The mobile phase was a gradient of water with acetic acid at 0.1% (*v*/*v*) (solvent A) and acetonitrile with acetic acid at 0.1% (*v*/*v*) (solvent B) as described by [53] with modifications. The elution gradient used was as follows: 0.00 min, 100% A; 1.00 min, 90% A and 10% B; 7.10 min: 75% A and 25% B; 25.00 min: 60% A and 40% B; 27.00 min: 100% B; 30.00, 100%. The injection volume was 1 μL, the flowrate of the mobile phase was 0.4 mL/min, and the temperature of the analytical column was 20 °C. The quantification of phenols was performed by using external standards in the range of 10 to 500 μM.

### 4.6. Statistical Analysis

Data are presented as mean ± standard deviation (SD) from two independent experiments, each comprising two biological replicates of five pooled shoots. Statistical analyses were conducted using the SPSS software (version 28.0, IBM Corp., Armonk, NY, USA). One-way and two-way analyses of variance (ANOVA) were conducted, as appropriate, to assess the significance of treatment effects. When one-way ANOVA indicated significant differences, Tukey’s post hoc test (*p* ≤ 0.05) was applied. Statistically significant effects identified by two-way ANOVA were attributed to M (basal medium composition), S (culture system), or M × S (interaction between medium composition and culture system). All graphs were generated using R statistical software (version 4.3.1; https://www.R-project.org/ (accessed on 15 November 2025)).

## Data Availability

The original contributions presented in this study are included in the article/Supplementary Material. Further inquiries can be directed to the corresponding author.

## Supplementary Materials

The following supporting information can be downloaded at: https://www.mdpi.com/article/10.3390/plants14243863/s1, Table S1. Mineral nutrient composition of the four basal media formulations used in this study; Table S2. Two-way ANOVA results for the effects of basal media composition (M), culture system (S) and their interactions (M × S); Figure S1. Pearson correlation coefficients among physiological and biochemical parameters measured in *in vitro*-grown spearmint shoots cultured in four basal media formulations (MS, MS/2, M/2S, and MSN/2) under three culture systems (agar-solidified, static liquid, and agitated liquid media); Figure S2. *In vitro* spearmint shoots cultured in half-strength Murashige and Skoog (MS/2) media under three culture systems—agar-solidified, static liquid, and agitated liquid—after 4 weeks (**a**). Representative close-up images of spearmint shoots cultivated in each culture system (**b**).

## Abbreviations

The following abbreviations are used in this manuscript:

AMY	α-amylase inhibitory activity
chl	Chlorophyll
DPPH	DPPH radical scavenging activity
MDA	Malondialdehyde
MS	Full-strength Murashige and Skoog medium
MS/2	Half-strength MS medium
M/2S	MS with diluted (1/2) macronutrients and iron elements
MSN/2	MS with diluted (1/2) nitrate and ammonium concentration
RA	Rosmarinic acid
STA	Starch content
TFC	Total flavonoid content
TPC	Total phenol content
TSS	Total soluble sugars
