# Supplementary material for: Culture System and Nutrient Restriction Shape Antioxidant Activity in In Vitro Spearmint (Mentha spicata L.) Shoots
Source: Plants (Basel). 2025 Dec 18;14(24):3863. doi: 10.3390/plants14243863 (PMC12736703; doi:10.3390/plants14243863)

Culture System and Nutrient Restriction Shape Antioxidant Activity in *in vitro* Spearmint (*Mentha spicata* L.) Shoots

Raquel Martínez-Carrillo‡, Fátima Z. Behloul‡, María Á. Ferrer, Antonio A. Calderón*

Supplementary material

Table S1. *Mineral nutrient composition of the four basal media formulations used in this study.* ***2***

Table S2. *Two-way ANOVA results for the effects of basal media composition (M), culture system (S) and their interactions (MxS).*... ***3***

Figure S1. *Pearson correlation coefficients among physiological and biochemical parameters measured in in vitro spearmint shoots cultured in four basal media formulations (MS, MS/2, M/2S and MSN/2) under three culture systems (agar-solidified, static liquid, and agitated liquid media).* ***4***

Figure S2. *In vitro spearmint shoots cultured in half-strength Murashige and Skoog (MS/2) media under three culture systems —agar-solidified, static liquid, and agitated liquid— after 4 weeks (a). Representative close-up images of spearmint shoots cultivated in each culture system (b).* ***5***

Table S1. Mineral nutrient composition of the four basal media formulations used in this study.

Abbreviations: MS, full-strength Murashige and Skoog medium; MS/2, half-strength MS medium; M/2S, MS with diluted (1/2) macronutrients and iron elements; MSN/2, MS with diluted (1/2) nitrate and ammonium concentration.

Element (mM)	MS	MS/2	M/2S	MSN/2
N (NO_3^-)	39.401	19.700	19.700	19.700
N (NH_4^+)	20.610	10.305	10.305	10.305
P	1.250	0.625	0.625	1.250
K	20.045	10.023	10.035	10.650
S	1.731	0.865	0.215	1.730
Ca	2.990	1.495	1.495	2.990
Mg	1.500	0.750	0.750	1.500
Fe	0.100	0.050	0.050	0.100
Cl	5.983	2.991	2.990	5.983
B	0.100	0.050	0.100	0.100
Mn	0.100	0.050	0.100	0.100
Zn (µM)	29.91	14.96	29.91	29.91
Mo (µM)	1.00	0.50	1.00	1.00
Cu (µM)	0.10	0.05	0.10	0.10
Na	0.202	0.101	0.202	0.202
I (µM)	5.00	2.50	5.00	5.00
Co (µM)	0.11	0.06	0.11	0.11

Table S2. Two-way ANOVA results for the effects of basal media composition (M), culture system (S) and their interactions (MxS).

Asterisks indicate the level of significance (*$p \leq 0.05$, **$p \leq 0.01$, *** $p \leq 0.001$, ns: not significant).

	Media composition (M)			Culture system (S)			Interaction MxS		
	df	F	*p*-value	df	F	*p*-value	df	F	*p*-value
**AMY**	3	73.475	2.0 e $^{-15}$ ***	2	82.174	3.8 e $^{-14}$ ***	6	15.957	7.5 e $^{-9}$ ***
***chl*a**	3	1.969	0.136 ns	2	1.433	0.252 ns	6	1.002	0.439 ns
***chl*b**	3	3.044	0.041 ns	2	4.683	0.016 ns	6	1.606	0.174 ns
**DPPH**	3	241.828	6.6 e $^{-24}$ ***	2	591.055	2.9 e $^{-28}$ ***	6	91.003	2.9 e $^{-20}$ ***
**FRAP**	3	63.366	1.9 e $^{-14}$ ***	2	111.106	3.9 e $^{-16}$ ***	6	19.054	7.6 e $^{-10}$ ***
**MDA**	3	4.703	0.007 **	2	13.211	4.9 e $^{-5}$ ***	6	2.299	0.056 ns
**RA**	3	648.631	2.2 e $^{-31}$ ***	2	911.379	1.4 e $^{-31}$ ***	6	316.679	1.2 e $^{-29}$ ***
**STA**	3	36.297	5.5 e $^{-11}$ ***	2	61.315	2.5 e $^{-12}$ ***	6	18.520	1.1 e $^{-9}$ ***
**TFC**	3	108.185	4.5 e $^{-18}$ ***	2	194.539	5.0 e $^{-20}$ ***	6	37.160	5.3 e $^{-14}$ ***
**TPC**	3	54.461	1.8 e $^{-13}$ ***	2	107.507	6.5 e $^{-16}$ ***	6	14.506	2.4 e $^{-8}$ ***
**TSS**	3	30.665	5.0 e $^{-10}$ ***	2	65.574	9.9 e $^{-13}$ ***	6	7.518	2.8 e $^{-5}$ ***

Figure S1. Pearson correlation coefficients among physiological and biochemical parameters measured in *in vitro* spearmint shoots cultured in four basal media formulations (MS, MS/2, M/2S and MSN/2) under three culture systems (agar-solidified, static liquid, and agitated liquid media).

Abbreviations: AMY, α-amylase inhibitory activity; chl, chlorophyll; DPH, DPPH radical scavenging activity; FRAP, ferric reducing antioxidant power; MDA, malondialdehyde; RA, rosmarinic acid; STA, starch content; TFC, total flavonoid content; TPC, total phenol content; TSS, total soluble sugars.

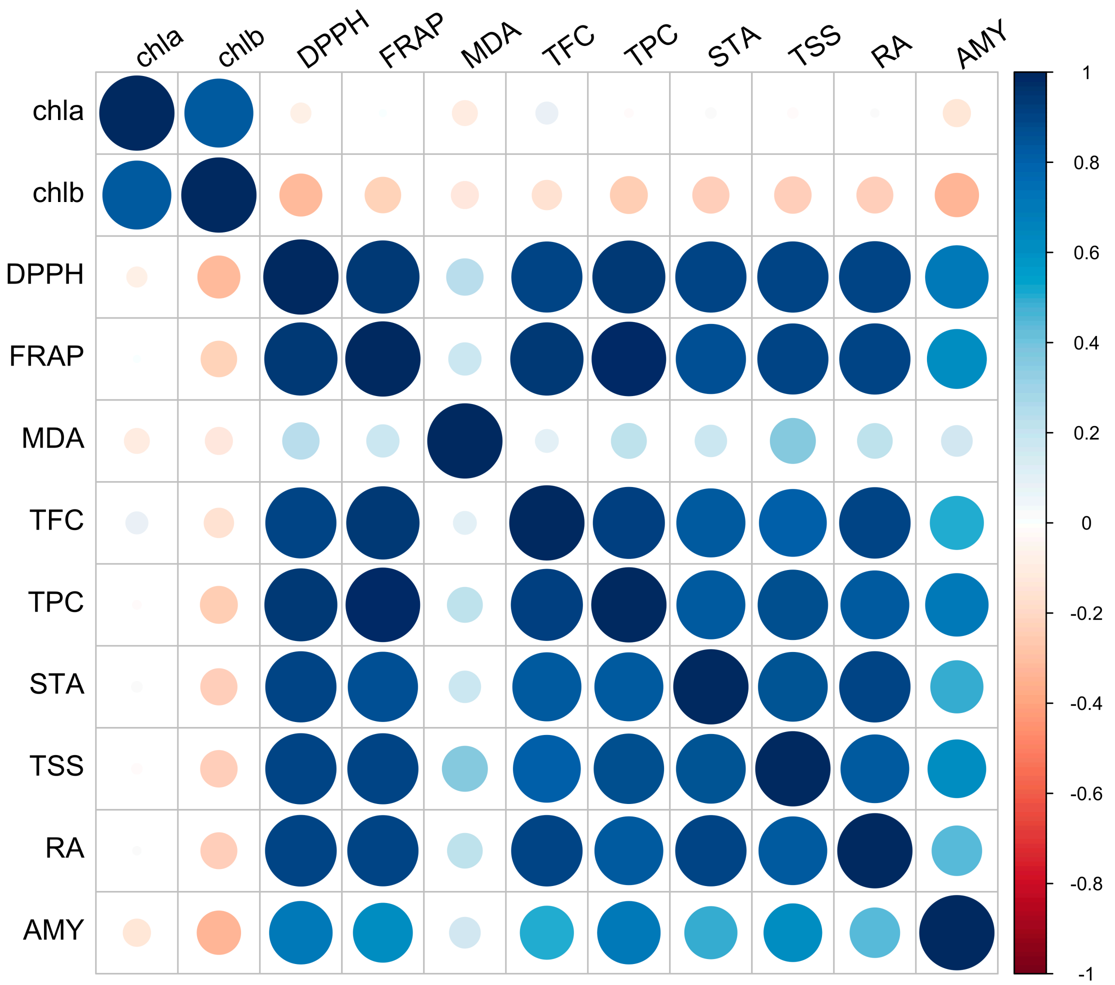

Figure S2. *In vitro* spearmint shoots cultured in half-strength Murashige and Skoog (MS/2) media under three culture systems —agar-solidified, static liquid, and agitated liquid— after 4 weeks (**a**). Representative close-up images of spearmint shoots cultivated in each culture system (**b**).

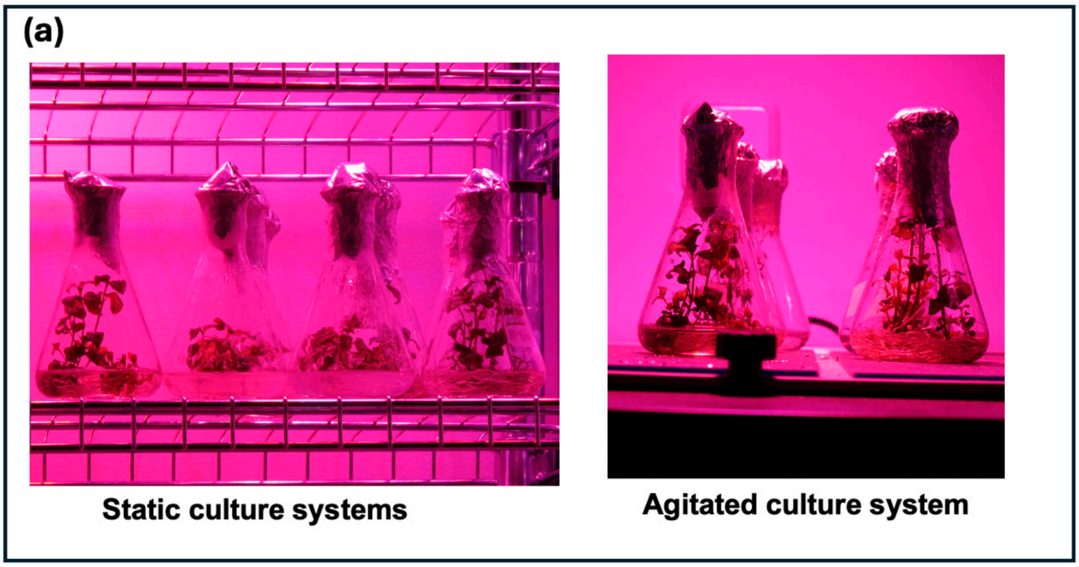

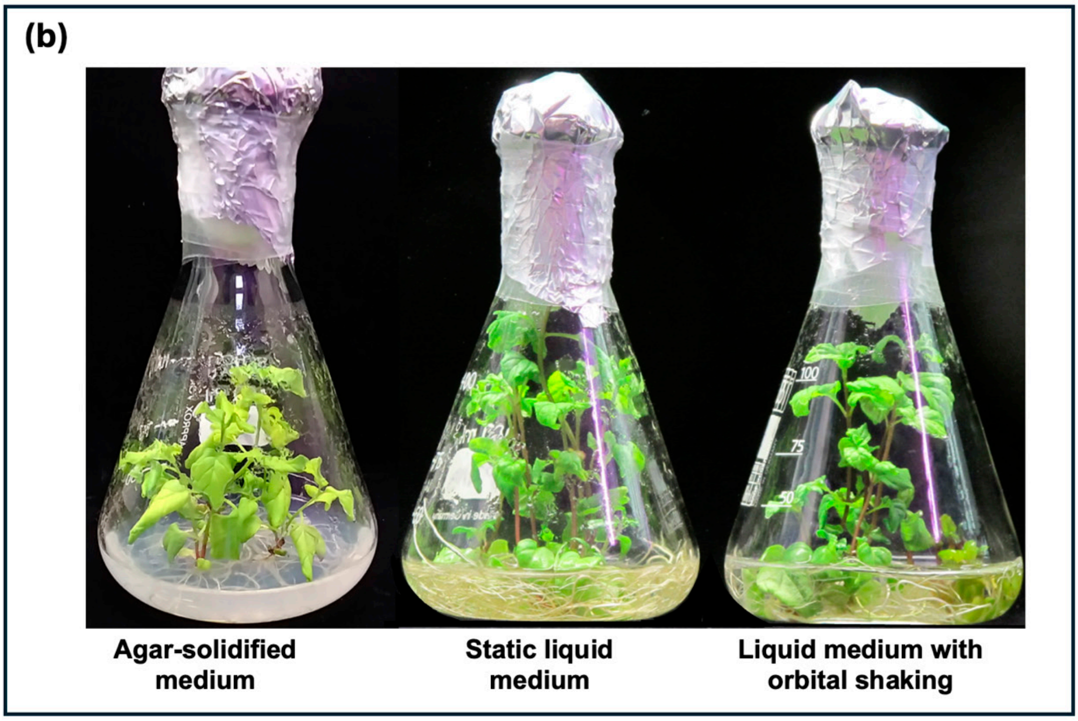

Supplement: Supplementary file 1 [file plants-14-03863-s001.zip › plants-4018581-supplementary.pdf]
